# Migration and standing variation in vaginal and rectal yeast populations in recurrent vulvovaginal candidiasis

**DOI:** 10.1128/msystems.00157-25

**Published:** 2025-09-04

**Authors:** Abdul-Rahman Adamu Bukari, Rebekah J. Kukurudz-Gorowski, Alexia de Graaf, Devin A. Habon, Beamlak Manyaz, Yana Syvolos, Aruni Sumanarathne, Vanessa Poliquin, Aleeza C. Gerstein

**Affiliations:** 1 Department of Microbiology, Faculty of Science, University of Manitoba 468335, Winnipeg, Manitoba, Canada; 2 Obstetrics, Gynecology and Reproductive Sciences, University of Manitoba 8664, Winnipeg, Manitoba, Canada; 3 Department of Statistics, Faculty of Science, University of Manitoba 8664, Winnipeg, Manitoba, Canada; University of Massachusetts Amherst, Amherst, Massachusetts, USA

**Keywords:** recurrent vulvovaginal candidiasis, yeast infection, *Candida albicans*, *Nakaseomyces glabratus*, yeast genomics

## Abstract

Vulvovaginal candidiasis is a very common human fungal infection. Most are successfully treated with antifungal drugs, yet ~8% lead to recurrent vulvovaginal candidiasis (RVVC). Vaginal and rectal populations have been previously found to be closely related in RVVC. Yet the genomic methods used precluded the assessment of fine-scale relationships and the measurement of within-population variation, a fundamental property with evolutionary implications. To address this gap, we isolated 12 vaginal and 12 rectal yeast isolates from four individuals with a history of RVVC. Three individuals had *Candida albicans* infections*,* while the fourth had *Nakaseomyces glabratus*. All isolates were whole-genome sequenced and phenotyped. Isolates were placed into species-level phylogenies composed of isolates from many different countries and contexts, including an updated *N. glabratus* tree with over 500 isolates. Genotypic and phenotypic analyses were consistent with migration between sites. There was little phenotypic diversity in drug response and no consistent difference between isolates from different sites for invasive growth. Although there are few comparables, *C. albicans* nucleotide diversity was similar to most commensal oral and rectal populations, while *N. glabratus* was similar to some bloodstream infections (though higher than others). Single-nucleotide changes drove intra-population genetic differences; only a single loss-of-heterozygosity tract varied among isolates from within one participant. This study provides baseline measurements and describes techniques to quantify within-population diversity in fungal microbes. We highlight a need for comparable studies that use the same sampling effort and analysis methods to understand the interplay between evolutionary factors in shaping fungal microbial communities.

**IMPORTANCE:**

Vaginal yeast infections are very common, yet we do not understand why some people experience chronic infections when most have a single infection that is successfully treated and cleared. We examined 12 vaginal and 12 rectal yeast isolates from four individuals with a history of recurrent yeast infections (total 96 isolates). Three participants had a *Candida albicans* infection (the most common causative species), while the fourth had a *Nakaseomyces glabratus* infection (the second most common and increasingly implicated). We found that vaginal and rectal isolates were closely related genetically, indicating the same population is present at the two sites. Surprisingly, we found that diversity was similar to the yeast populations found at other body sites in healthy people. Our study highlights a critical need for additional studies following the same methods in different contexts to better understand the fungal microbial populations in our bodies.

## INTRODUCTION

Vulvovaginal candidiasis (VVC; colloquially “yeast infection”) is common, affecting approximately 75% of people defined female at birth at least once in their lives (1–3). The disease burden of VVC results in global annual treatment costs of ~1.8 billion USD (4, 5), with a loss in productivity in high-income countries of ~14 billion USD (4). Treatment involves topical or oral antifungal medication, which is effective at symptom abatement in most cases. However, ~8% of individuals with VVC experience recurrence (recurrent vulvovaginal candidiasis [RVVC]), defined as three or more symptomatic episodes a year (4, 5). Co-morbidities that involve the vaginal microbiome, such as recurrent bacterial vaginosis (6) or frequently taking antibiotics to treat conditions such as cystic fibrosis (7), are known to predispose individuals to RVVC. Similarly, treatment for conditions that lead to alteration of the vaginal mucosa, such as hormone replacement therapy in postmenopausal women (8), has also been associated with increased prevalence, as has a small number of human genetic variants such as mannose-binding lectin deficiency (9, 10) and TLR2 Pro631His polymorphism (11). Yet, approximately half of all people with RVVC have no identifiable risk factors (12), signifying the need for additional studies on the biological basis of this chronic condition.

*Candida albicans* is responsible for 50%–90% of VVC and RVVC cases (collectively, R/VVC) (13–17). *Nakaseomyces glabratus* (formerly *Candida glabrata*) is the second most prevalent cause, globally attributed to ~8% of cases (18–20). Here, we collectively refer to these species using the colloquial term “yeast,” which reflects a shared morphology while acknowledging our current understanding of their divvergent phylogenetic relationships and the recent official renaming of *N. glabratus* away from *Candida glabrata* (21, 22). To be consistent with clinical practice, we continue to use the R/VVC abbreviations while noting that “candidiasis” does not reflect the updated genus names. Treatment recommendation for R/VVC differs by species, as many isolates from *N. glabratus* (and other non-*albicans* pathogenic yeast species) have intrinsic resistance to the azole antifungal fluconazole (FLC) that is commonly used to treat RVVC (23).

Understanding the etiology of R/VVC is complicated in part since yeast are a common commensal member of the vaginal microbiota without causing symptomatic VVC (24), and *C. albicans* studies repeatedly find no strict phylogenetic differentiation between commensal and pathogenic strains. Relapse in RVVC, i.e., return of symptoms, could theoretically be due to either incomplete eradication of the vaginal yeast population after taking antifungal drugs or complete vaginal eradication followed by re-colonization (25). Decades of studies have sought to understand the etiology of RVVC, as the answer has potential implications for improving treatment and reducing or eliminating symptom recurrence. The gastrointestinal (GI) tract has been suggested as a possible endogenous source population, yet a study in 1979 that treated RVVC patients with oral nystatin (NYT) to reduce the resident GI population found that it did not decrease the time to recurrence (26). Furthermore, studies examining yeast colonization of the GI tract through feces or rectal swabs during symptomatic recurrence find that not all participants are culture positive (26–32). However, this does not necessarily preclude that a small GI population is present in all individuals (below the culture detection limit in some), which could act as an endogenous reintroduction source under the right host conditions. Examining the diversity of strains at different body sites and among recurrent infections can potentially differentiate between relapse scenarios. If vaginal isolates are closely related to GI tract/rectal isolates but less diverse, that would be consistent with reintroduction. If vaginal isolates sampled at different time points consistently have the same genotype that is not present in the GI tract/rectal isolates, this would be consistent with incomplete eradication. Looking at the level of diversity and relationships among isolates acquired within a single time point (“standing variation”) at different body sites can also help us understand the adaptive potential and migration dynamics of yeast populations within the body.

Multilocus sequence typing (MLST) has been commonly used for phylogenetic analyses in the context of R/VVC (17, 33, 34). The overarching results are generally consistent with the maintenance of genotypes between symptomatic recurrences, with a few examples of novel genotypes arising at one time point compared to another. However, typically only one or a small number of isolates are examined at a given time. Thus, standing genetic variation could have been undetected. Only a single study sequenced two vaginal isolates from different time points using short-read whole-genome sequencing (WGS), and no previous studies have employed WGS to compare multiple isolates from the same time point. WGS removes the need to rely on a single or small number of markers, which could over- or underinflate the actual level of diversity. For example, Sitterlé et al. showed that while MLST revealed occasional differences among *C. albicans* oral isolates collected from three healthy individuals, whole-genome sequencing revealed that the three examined isolates were actually closely related in each case (35).

Standing genetic variation of yeast populations has only been quantified in a handful of contexts. Two studies in *C. albicans* that whole-genome sequenced 3–6 oral and rectal isolates from six healthy individuals found that although isolates were closely related in most cases, they differed by numerous single-nucleotide polymorphisms (SNPs), primarily resulting from short-range loss-of-heterozygosity (LOH) tracts (35, 36). Two individuals were, however, simultaneously colonized with oral isolates from different clades (36). A single *N. glabratus* study sequenced up to 10 isolates from nine patients with bloodstream candidemia (37); a pairwise SNP analysis was also consistent with closely related isolates. Variation in RVVC has yet to be quantified through WGS; hence, whether it is similar to commensal populations is unknown. It has also not been determined how many isolates should be sequenced to capture population diversity accurately.

Here, we build on previous work by conducting WGS paired with high-throughput phenotyping to quantify vaginal and rectal standing variation in participants with a history of RVVC when high vaginal and rectal yeast population sizes were observed. We compared our results to the few comparable studies that conducted WGS of contemporaneous yeast isolates from other contexts and statistically evaluated the minimum number of isolates required to accurately measure genetic variation. We obtained isolates from the same time point from four individuals with a history of RVVC. Three participants had *C. albicans* infections, and one had *N. glabratus*. From all individuals, we found a complete phylogenetic overlap of vaginal and rectal isolates and no reliable difference in phenotypes, consistent with high levels of migration. We found no evidence that diversity in populations from the two sites was different; levels of standing genetic variation were generally similar to what has been observed in other contexts. This suggests that despite frequent population bottlenecks caused by drug treatment, vaginal yeast diversity is maintained or rapidly restored.

## RESULTS

### THRIVE-yeast isolates in the global species phylogenies

Participants with a history of RVVC were recruited from a specialty yeast clinic in Winnipeg, Canada. Vaginal and rectal isolates were collected from swab elutes plated onto Sabouraud dextrose agar(SDA) and chromogenic *Candida* agar from seventeen participants who were enrolled and screened intermittently between January 2020 and November 2022. As the goal was to quantify standing genetic variation from single time point vaginal and rectal populations, we haphazardly isolated vaginal and rectal yeast isolates from each of the four participants where we had at minimum 12 isolates from each site, for a total of 96 isolates. We refer to these isolates as the “THRIVE-yeast” isolates, following the name of our local umbrella research program that studies The Host-microbial Relationships and Immune function in different Vaginal Environments (THRIVE; http://www.mthrive.ca). Isolates from one participant were *N. glabratus* (YST6), while *C. albicans* was isolated from the other three (TVY4, TVY10, and YST7). Three isolates (TVY10R13, TVY4R4, and YST7R13) had low depth of sequencing coverage (<20×) and were excluded from the genomic but not phenotypic analyses. All *C. albicans* isolates were MAT-heterozygous diploids (a/α), while the *N. glabratus* isolates were all MTL1a.

**Fig 1:**
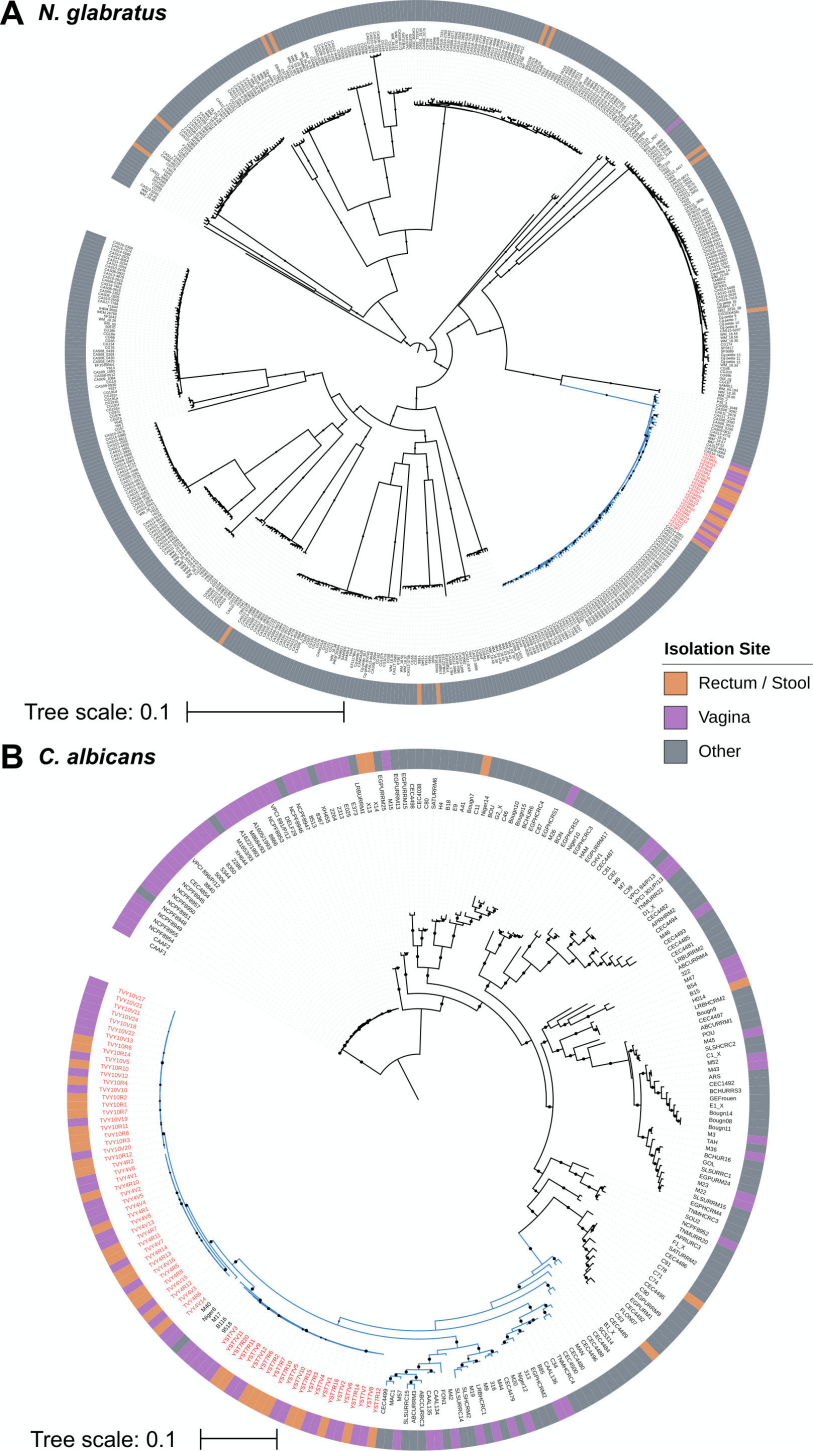
Approximate maximum likelihood phylogenies of (**A**) *N. glabratus*, including 526 global isolates, and YST6 vaginal and rectal isolates, and (**B**) *C. albicans*, including 182 isolates from reference 38 and vaginal and rectal isolates from TVY4, TVY10, and YST7. THRIVE-yeast isolates are indicated with red labels. The YST6 *N. glabratus* isolates are in a cluster of ST16 isolates, while the TVY4, TVY10, and YST7 *C. albicans* isolates are in clade 1 (blue branches indicate [A] ST16 and [B] clade 1 isolates). The *N. glabratus* phylogeny was rooted at the midpoint, and the *C. albicans* tree was rooted by *Candida africana* isolates (gray labels).

The phylogenetic relationship of the THRIVE-yeast isolates was evaluated in the context of available short-read WGS isolates from each species. We exhaustively searched the National Center for Biotechnology Information (NCBI) for available *N. glabratus* sequences, finding and downloading fastq data from 526 isolates (Table S1). Notably, we only found a single annotated vaginal isolate and 12 stool isolates, while over 80% of the isolates are blood isolates. We combined the fastq data from the 24 YST6 isolates and 526 global isolates to construct the largest *N. glabratus* phylogenetic tree to date. The YST6 isolates are monophyletic and cluster with 68 bloodstream isolates and three isolates of unknown provenance from the United States, Canada, and Australia (Fig. 1A).

The widely used *C. albicans* phylogeny is composed of 182 isolates from a wide breadth of geographic and anatomical sites (38). TVY4, TVY10, and YST7 isolates all form intra-population monophyletic groups that group together in a subgroup that contains 23 additional isolates in clade 1, the most common clade (Fig. 1B). TVY4 and TVY10 isolates are beside each other and shared a common ancestry with M40, which is also a vaginal isolate from Morocco. The YST7 isolates are most closely related to three vaginal isolates (one each from Brazil, Morocco, and China) and one oral isolate from Niger. Seven of the remaining 18 isolates in the clade 1 subgroup were also isolated from the vagina. This is a statistical enrichment for vaginal isolates compared to the rest of the isolates in clade 1 (THRIVE-yeast isolates from each participant were counted as a single isolate; Fisher exact test comparing 14 vaginal isolates out of 26 in the subgroup to 3 vaginal isolates out of 17 in the rest of clade 1, *P* = 0.026). If we discount the 35 predominantly vaginal isolates in clade 13, which is now recognized as likely a separate species (*Candida africana)* (39, 40), clade 1 as a whole is also statistically overrepresented for vaginal isolates compared to the *C. albicans* tree in general (17 vaginal isolates in clade 1 out of 43 total isolates, compared to 18 vaginal isolates out of 107 total isolates; Fisher exact test, *P* = 0.005). Thus, although the sequenced vaginal isolates are located in six different clades, they are over-represented in clade 1 relative to a neutral expectation that vaginal isolates are equally likely to be found anywhere in the existing tree.

### Vaginal and rectal isolates are closely related and phylogenetically overlapping

**Fig 2:**
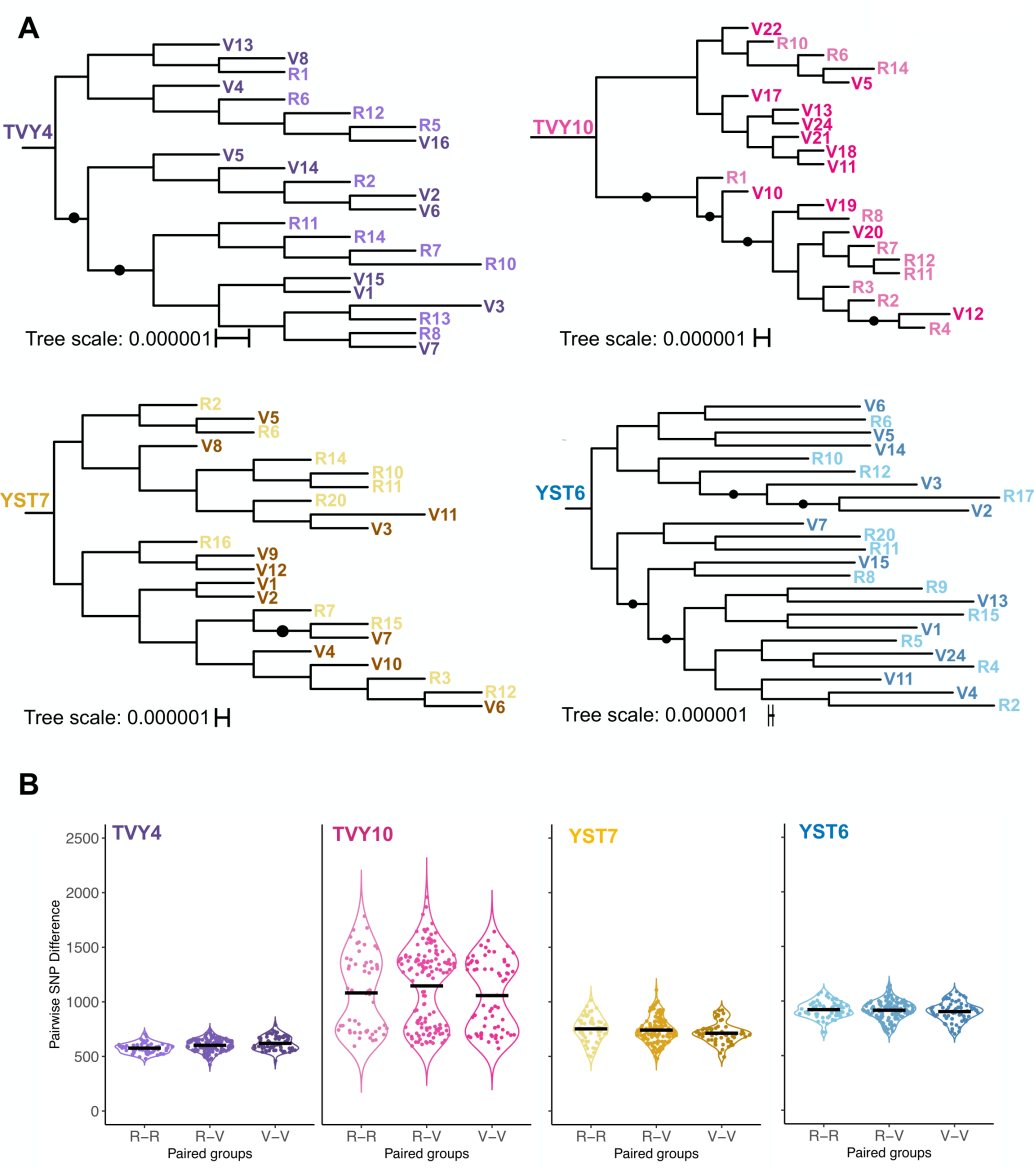
Within participant phylogenetic and SNP analyses. (**A**) The fine-scale phylogenetic structure among THRIVE-yeast isolates shows that vaginal and rectal isolates are closely related and do not segregate by site of isolation. Vaginal (V) and rectal (R) isolates were acquired from four participants with a history of RVVC (YST6: *N. glabratus*; TVY4, TVY10, and YST7: *C. albicans*). The isolate numbers are arbitrary based on the order in which they were collected from culture plates. Black circles indicate branches with bootstrap support ≥0.8. (**B**) Within-participant pairwise comparison of SNPs between isolates from the indicated sites (V-V: vaginal-vaginal, R-V: rectal-vaginal, and R-R: rectal-rectal) confirms that SNPs are distributed evenly among vaginal and rectal isolates.

Following the observed monophyly among isolates from the same participant, we next assessed the relatedness of vaginal and rectal isolates. We first generated phylogenies for each individual using RAxML (41). The vaginal and rectal isolates from all four participants are phylogenetically overlapping (Fig. 2A). Few branches within the four phylogenies had bootstrap support exceeding 80%, yet well-supported clusters in all participants included isolates from both body sites. For each population, we also conducted a local principal component analysis (PCA) that examines the regions of high genomic heterogeneity within populations. From all participants, the regions of high differences were generally distributed throughout the genome, and the PCA failed to segregate vaginal and rectal isolates (Fig. S1).

### Pairwise differences in single-nucleotide polymorphisms among isolates per participant

Although isolates were closely related, WGS data identified SNP differences among all pairs of participant isolates. To test whether within-population vaginal diversity was lower than within-population rectal diversity, we compared pairwise SNP differences among vaginal isolates to the pairwise SNP differences among rectal isolates. To test whether there was a signal of divergence between sites, we also compared single-site differences to pairwise differences between isolates across sites. The average pairwise SNP differences between vaginal isolates were very similar to average pairwise SNP differences between rectal isolates and between isolates from different sites (Fig. 2B). The only significant difference was in TVY4, where the average SNP differences among rectal isolates were significantly lower than the vaginal isolates (analysis of variance [ANOVA] test, YST7: F_2, 250_ = 2.164, *P* = 0.117; YST6: F_2, 250_ = 0.785, *P* = 0.457; TVY4: F_2, 250_ = 8.599, *P* = 0.000244; TVY10: F_2, 250_ = 1.66, *P* = 0.192; Tukey’s honest significance test [HSD] test for multiple comparisons; *P* = 0.0001). The distributions were fairly normal, as expected for isolates with low population structure, except that TVY10 isolates showed a bimodal distribution of pairwise SNP differences between isolates from both the vaginal and rectal sites.

### Minimum number of isolates for estimating standing genetic variation within participants

**Fig 3:**
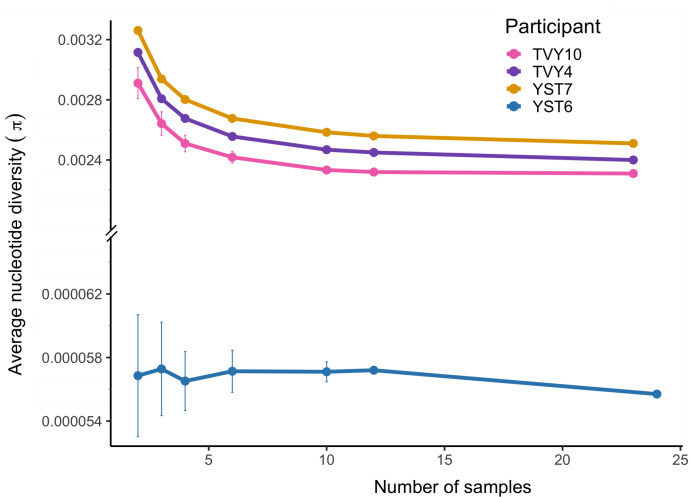
Calculated nucleotide diversity decreases, then plateaus with an increasing number of samples. π was calculated for different numbers of samples from each individual. For *n* = 2, 3, 4, 6, or 10, the given number of samples was randomly selected from the vaginal isolate set. For *n* = 12, the data sets were generated by randomly choosing six samples from each of the rectal and vaginal isolate sets. The bootstrap analysis was done 50 times for each sample size, and the mean and SD among data sets were calculated. For *n* = 23 (*C. albicans*, TVY10, TVY4, and YST7) or 24 (*N. glabratus*, YST6), the nucleotide diversity of all samples was calculated.

A major goal of our work was to compare diversity within RVVC populations to diversity observed in other contexts to make inferences about the evolutionary process based on the observed degree of standing genetic variation. However, the small number of comparable studies sequenced different numbers of isolates, and nucleotide diversity (π) will decrease with an increased number of samples taken from a population. To quantify the scale of this effect of changing the number of isolates, we conducted a bootstrap analysis using our 12 vaginal isolates from each individual. We repeatedly resampled 3–10 isolates and recalculated diversity. For all individuals, the shape of the diversity curve with the number of isolates was very similar—an elbow was observed around *n* = 6 (Fig. 3). Nucleotide diversity in YST6 (*N. glabratus*) was two orders of magnitude lower, and there was not a consistent change in diversity with the number of isolates.

### THRIVE-yeast isolates share similar diversity as isolates from commensal and other disease settings

**Fig 4:**
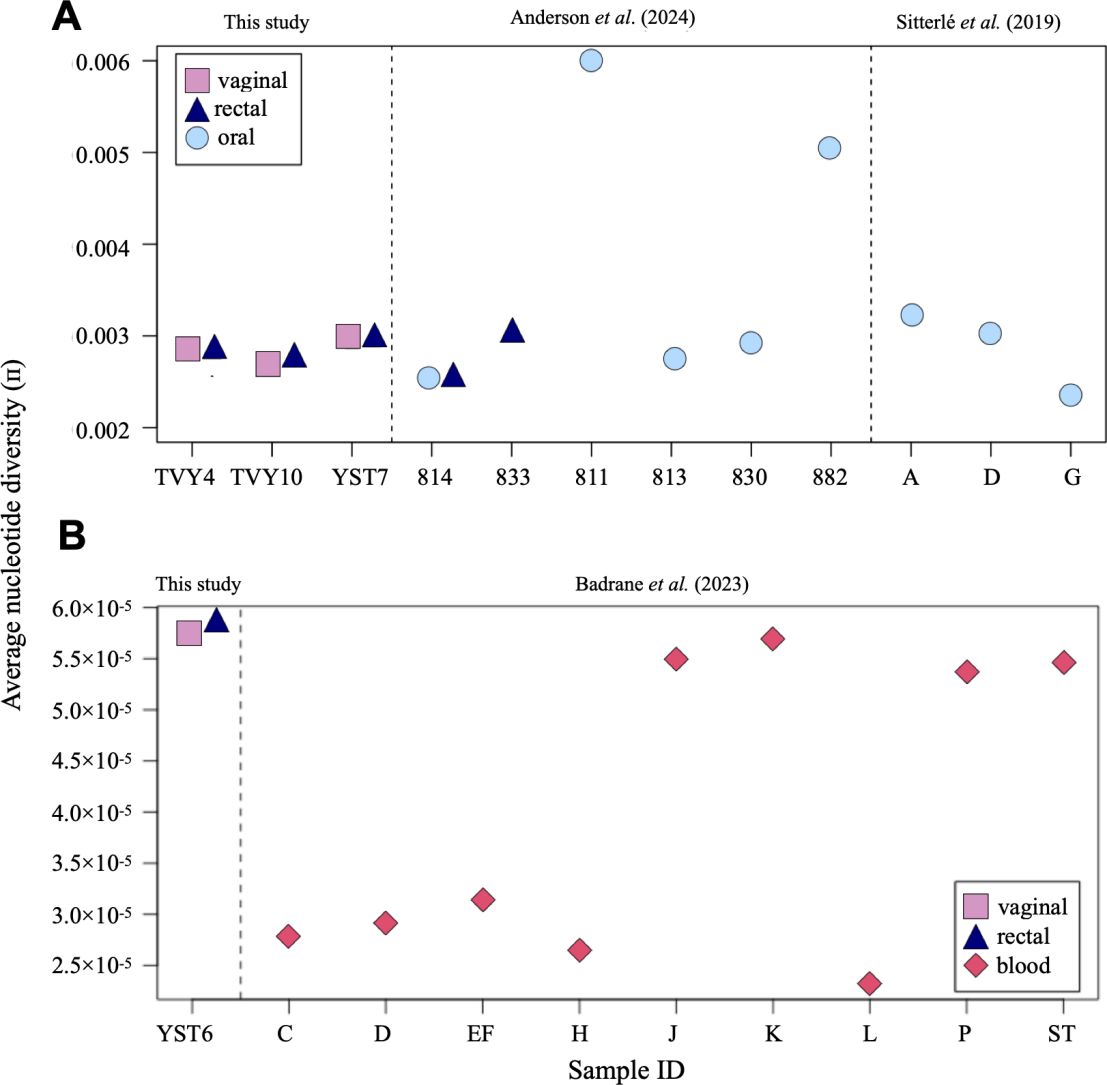
The average nucleotide diversity of vaginal and rectal populations from RVVC is similar to populations from other contexts. (**A**) Comparison of π in THRIVE *C. albicans* isolates from TVY4, TVY10, and YST7 to commensal isolates from two previously published studies. For accurate comparison, three randomly chosen samples were subsampled from each site in the THRIVE-yeast isolates. (**B**) Comparison of π in THRIVE *N. glabratus* (YST6) isolates to bloodstream infection isolates from 10 individuals in a previous study.

We downloaded the fastq files from two previous *C. albicans* studies on commensal populations (35, 36) and used our pipeline to calculate the average nucleotide diversity for each. For all populations, including our own, where necessary, we down-sampled the number of isolates to three, consistent with the lowest number of isolates sampled from the commensal populations. The average nucleotide diversity was very similar across most populations (Fig. 4A). The exception was two oral populations from two participants previously shown to have isolates from different phylogenetic clades. The YST6 vaginal isolate population was compared to fastq data from one previous *N. glabratus* study that examined 9–10 isolates from nine different bloodstream infection (BSI) populations (37). The average nucleotide diversity from YST6 vaginal and rectal populations was similar to the diversity from four participants yet much higher than the average nucleotide diversity from the other five (Fig. 4B). Interestingly, the four BSI populations with similar diversity to YST6 are all from the same multilocus sequence typing (ST) group,ST3, which is closely related to the cluster that contains YST6 (ST16). By contrast, the other populations are from more distantly related clades (37). Future work in *N. glabratus* should more deeply explore whether there is a consistent relationship between clade and within-population genetic diversity.

### Little variation in copy number or loss of heterozygosity within populations

**Fig 5:**
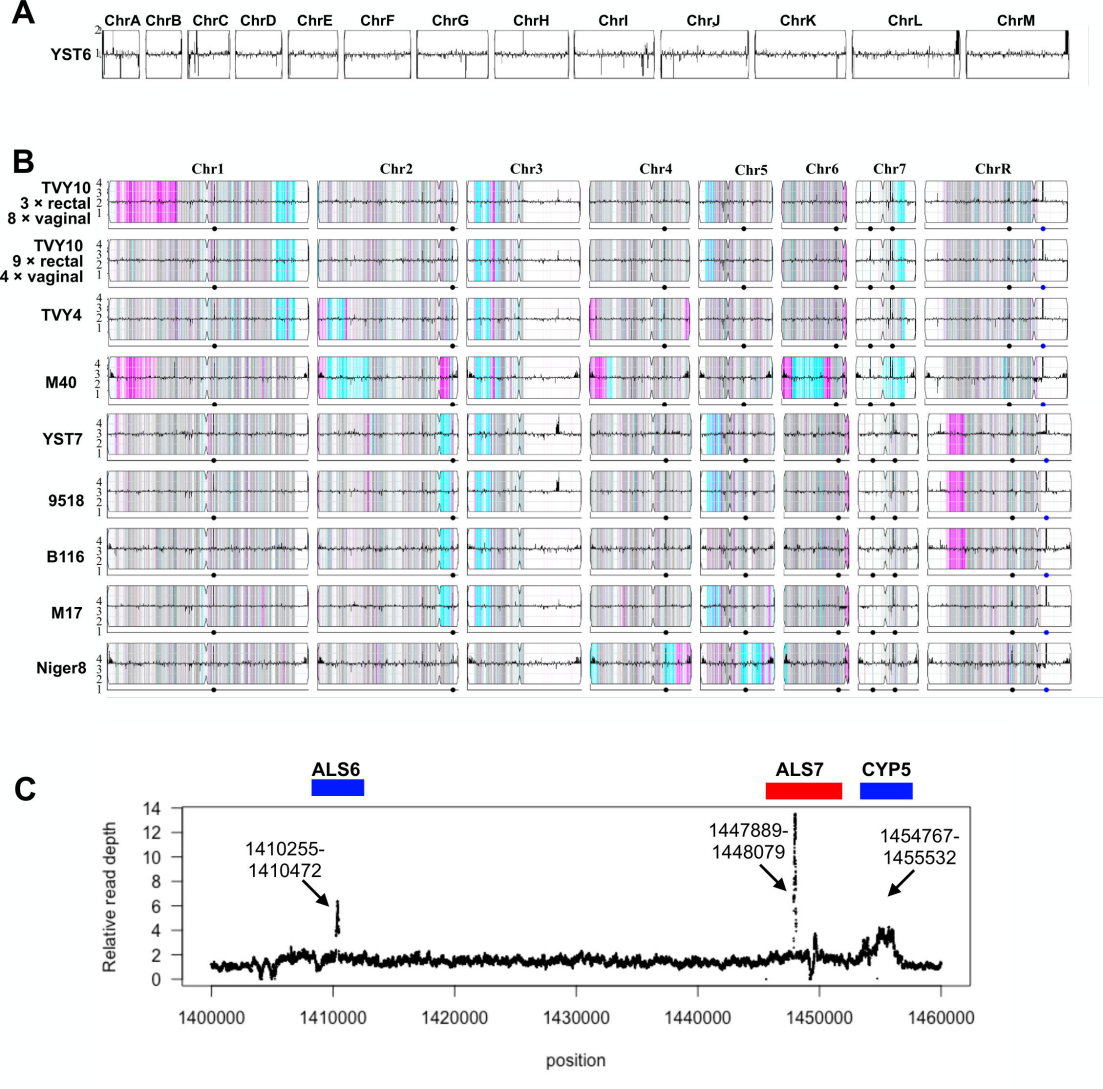
CNV and LOH profiles of THRIVE-yeast. Representative traces from (**A**) YST6 and (**B**) TVY10, TVY4, YST7, and five closely related isolates. All isolates are euploid, indicated by the horizontal black line in each panel, which indicates relative copy number by comparing the number of reads that map to each position compared to the reference genome. For *C. albicans* isolates in panel B, the density of heterozygous SNPs in 5 kb bins is shown as vertical colored lines. Regions with heterozygous SNPs are gray, and regions with homozygous SNPs are colored based on the retained SC5314 haplotype: cyan for “AA” and magenta for “BB.” White indicates an ancestral LOH in SC5314. For each chromosome, the centromere is indicated by an indentation in the box. The dots on the bottom line below each box indicate the positions of major repeat sequences. (**C**) Fine-scale coverage mapping of the putative CNV on chr3. Shown is one representative trace from YST7 R2; all isolates have a similar pattern. Gene positions above the figure are approximated.

We examined copy number variation (CNV) and LOH events among THRIVE-yeast isolates and their closest relatives using Y_MAP_ (42). No CNVs were identified in any YST6 isolates (Fig. 5A). A single ~50 kb CNV on the right arm of chr3 was identified in all YST7 isolates (Fig. 5B). This CNV is also present in the closest relative to the YST7 isolates, vaginal strain 9518, but is absent in the next two closely related strains that are also vaginal in origin (B116 and M17). As Y_MAP_ visualizations are based on averages across 5,000 bp sliding windows, we examined the region in finer detail. Coverage was measured from the binary alignment map (BAM) files to compute the depth at each position in that region. Mapped coverage was inconsistent with the profile of a typical CNV; the majority of the region had only a slightly elevated copy number relative to the rest of the genome (Fig. 5C). Two small (<200 bp) regions spiked up to approximately 6- and 14-fold coverage, the first internal to *ALS6* and the second to *ALS7* (Fig. 5C). A third region composed of elevated coverage maps to another gene with close homology to other genes in the genome, *CYP5*, a putative peptidyl-prolyl cis-trans isomerase (43). The region identified in Y_MAP_ is thus likely to primarily reflect an error in mapping rather than a true CNV with potential biological effects. No other CNVs or aneuploidies were identified in the other THRIVE-yeast isolates.

LOH analysis in the *C. albicans* populations was consistent with the phylogenetic analysis and diversity metrics; generally, all isolates from the same participant shared an LOH profile. The only exception was in TVY10, where an LOH tract on the left arm of chr1 was found in three rectal and eight vaginal isolates that clustered together phylogenetically (Fig. 5B). Some LOH regions were similar among isolates from different participants. All isolates exhibited LOH on chr3L. TVY10 and TVY4 shared a ~300 kb LOH on chr1R, and both have an LOH region on chr7R. TVY10 and YST7 shared LOH on chr5L, with distinct allelic profiles. YST7 shared LOH regions with related vaginal isolates (9518, B116, and M17) but not the oral isolate Niger8 on the right arm of chr2, the left arm of chr5, and the left arm of chrR. These common LOH regions hint at the possibility of repeated selection for homozygosity in these regions.

### Phenotypic variation

**Fig 6:**
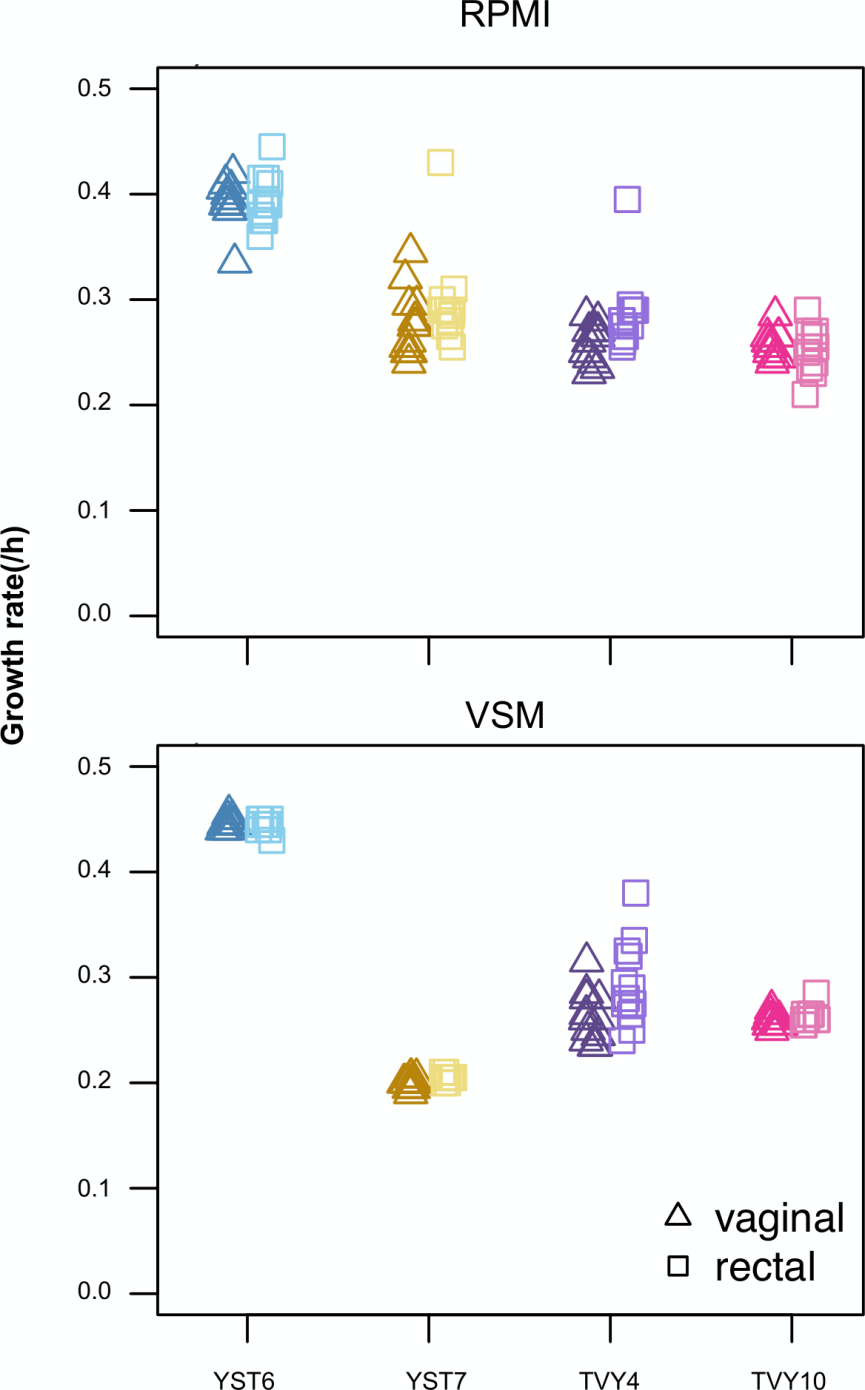
The growth rate was measured from 12 vaginal and 12 rectal isolates from each population. Optical density was recorded every 15 minutes in a plate reader with constant shaking and incubation at 37°C. Each point represents the mean of two technical replicates for each of the two biological isolates, and 24 isolates were measured for each group. The growth rate was calculated as the spline with the highest slope using a custom R script.

We quantified within-population phenotypic variation in parallel to genotypic variation. The average growth rate for YST6 *N. glabratus* isolates was higher than the *C. albicans* populations in both Roswell Park Memorial Institute (RPMI) medium (Fig. 6A) and vaginal simulative medium (VSM; Fig. 6B). Growth rates were either the same between vaginal and rectal isolates or the rectal isolates were higher when grown in either medium (Welch two-sample *t*-test [Table S4], rectal isolates higher in TVY4 grown in RPMI: *t* = 2.50, df = 16.1, *P*-value = 0.023; TVY4 grown in VSM: *t* = −2.36, df = 18.1, *P*-value = 0.030; YST7 grown in VSM: *t* = −3.63, df = 18.8, *P*-value = 0.002). Consistent with a visual inspection, single statistical rectal outliers with increased growth rate relative to other isolates were seen in YST7 and TVY4 grown in RPMI, and YST6 and TVY10 grown in VSM (Fig. 6, Rosner’s test for outliers). No additional outliers were identified in the other populations.

**Fig 7:**
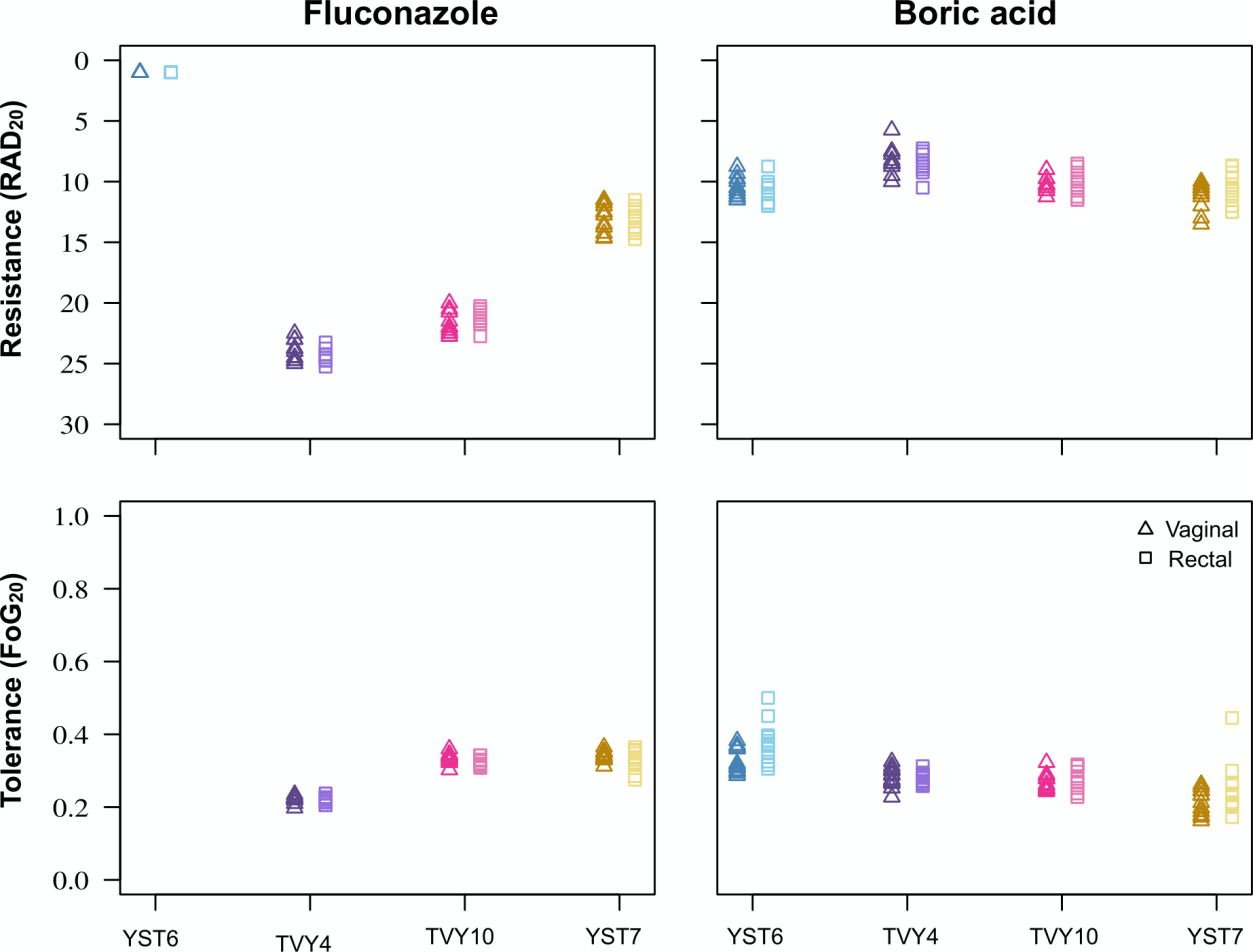
Little intra-population variation was found for drug response phenotypes measured from disk diffusion assays. Drug resistance (top panels) and drug tolerance (bottom panels) were measured for FLC and BA. Drug response was measured on pH 4 Mueller-Hinton plates using the R package diskImageR (47), which computationally measures response parameters from images of disk diffusion assays. Each point represents the mean of four replicates (two technical replicates for two biological replicates).

We then compared drug resistance and drug tolerance from vaginal and rectal isolates. We conducted a pilot experiment on 24 isolates from participant YST7 to quantify variation in drug resistance for five different drugs that are indicated as treatment options by the Society of Obstetricians and Gynaecologists of Canada for uncomplicated, recurrent, and non-albicans VVC (16). We also examined drug tolerance—the ability of drug-susceptible populations to grow slowly in the presence of high levels of fungistatic drugs—which also emerged as a trait that varies among different fungal species and isolates (44, 45). Tolerance may be implicated in the propensity to cause fungal disease in other contexts (46) but has not previously been examined in the context of R/VVC. We found very little variation among the isolates for either phenotype in any drug (Fig. S3). Given that, we proceeded with quantifying drug responses for just fluconazole and boric acid (BA) at pH 4.2, as these are drugs from different classes that are commonly prescribed in our local clinic. The site of isolation was only significant for YST6 BA resistance (vaginal isolates were slightly more tolerant than rectal isolates; *t*-test, *t* = 2.77, df = 18.2, *P*-value = 0.012; Table S4; Fig. 7). Formal outlier statistical tests that grouped vaginal and rectal isolates were broadly consistent with the qualitative visual assessment, identifying only two outlier isolates for tolerance (both rectal isolates in BA, one from YST6, one from YST7).

**Fig 8:**
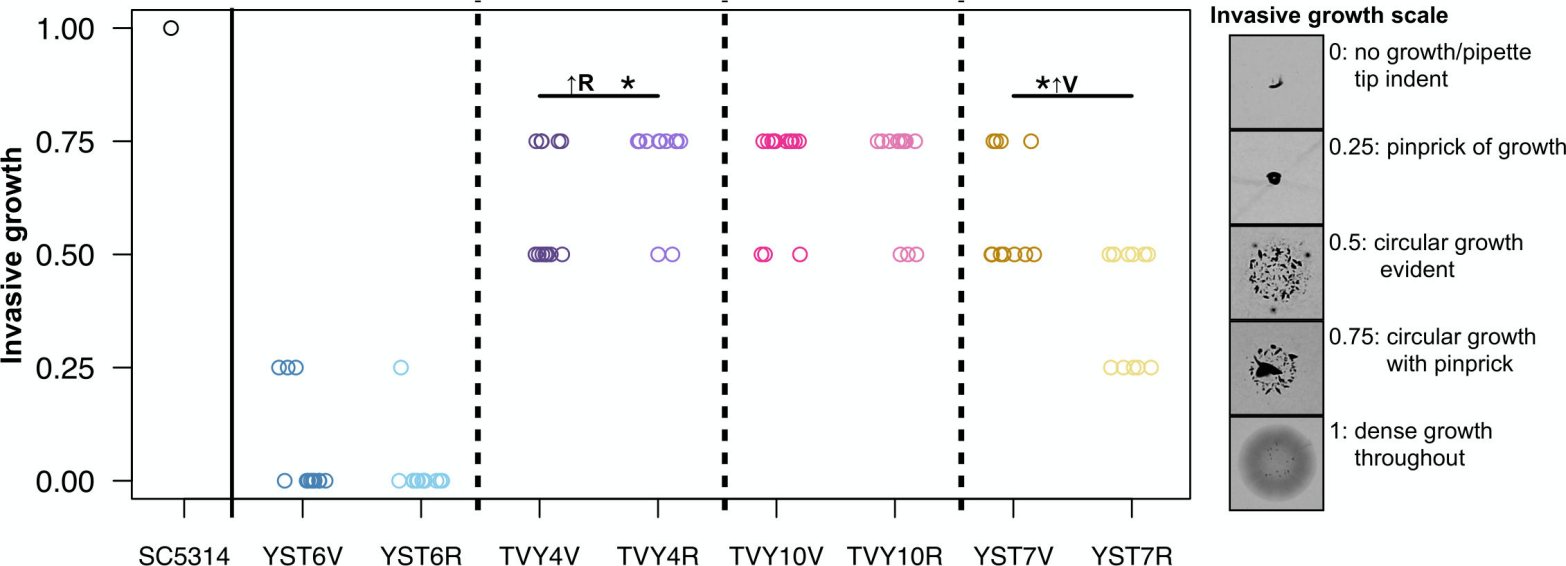
Invasive growth was qualitatively scored after growth on yeast peptone dextrose (YPD) plates for 96 h. Each point indicates the maximum score between two bio-replicates for each isolate.

There was considerable variation among participants and isolates for invasive growth (Fig. 8; Table S4). There was no difference between YST6 or TVY10 vaginal and rectal isolates, while YST7 vaginal isolates had higher invasive growth than rectal isolates (*W* = 115.5, *P* = 0.0002), and TVY4 rectal isolates exhibited higher invasive growth than vaginal isolates (*W* = 372, *P*-value = 0.032). The overall picture, thus, is that invasive growth seems to vary more between participants than between sites of isolation, and that statistical differences between sites are likely due to neutral processes rather than selection for invasive growth in the vaginal environment.

## DISCUSSION

The biological basis of RVVC requires further understanding. To study the vaginal yeast populations implicated in the disease, we quantified the diversity of 12 vaginal and 12 rectal isolates from four people with a history of RVVC who had large yeast populations at both sites at the time of sampling. In each case, the isolates formed monophyletic groups, and the vaginal and rectal isolates were phylogenetically overlapping, consistent with a common ancestral source and frequent migration between the two sites. This is in concordance with previous genetic studies that used more coarse sequencing methods, which found high genetic similarity between vaginal and rectal isolates in women with R/VVC (48–50). Our phenotypic analyses are also consistent with the genetic results; there was minimal diversity in drug responses or growth ability in clinically relevant medium and no consistent difference between isolates from different isolation sites for invasive growth. Previous studies have also found that virulence factor phenotypes have similar expression among vaginal and rectal isolates in the context of R/VVC (51) and among oral and rectal isolates from healthy individuals (36).

Multiple potential evolutionary explanations are consistent with our results. It is possible that the selective pressures most influencing adaptation are similar in both environments, leading to the selection of the same traits. However, it could also be that selection cannot overcome either (or both) migration or genetic drift due to a low effective population size. The isolates come from individuals with a long history of repeatedly taking antifungal drugs, and the yeast population sizes likely undergo many bottleneck cycles over time, functionally reducing the efficacy of selection. Nevertheless, we did observe hundreds of SNP differences between all isolate pairs, indicating the presence of what has been termed microvariation, which is potentially sufficient for adaptation.

The observed average nucleotide diversity among populations for the RVVC isolates was higher than expected. *A priori*, we predicted that genetic diversity would be highest in commensal populations from healthy individuals at sites that do not have obviously strong selective pressures acting on them and lowest in bloodstream infections, which are thought to be founded by a small population of circulating yeast cells. However, genetic diversity within the three RVVC *C. albicans* populations was similar to oral and rectal isolates from seven healthy individuals (though lower than two commensal oral populations previously known to have isolates from different clades) (35, 36). Given the limited sampling that has been conducted, it is difficult to make inferences about evolutionary dynamics and to benchmark “high” vs “low” diversity. There may be selection in the commensal oral (52) and rectal environments, involving the fixation of alleles and reducing genetic diversity to a similar degree as the RVVC populations. It could also be that the population bottlenecks in RVVC (and in at least some bloodstream infections) are not as strong as anticipated. Teasing apart these explanations will require intra-population data on many more populations from different contexts.

Importantly, we chose isolates for sequencing blind to phenotypic data to provide an unbiased estimate of genetic diversity. Although it is tempting to pick the most diverse isolates for in-depth genomic characterization, this makes it more difficult to compare results among studies (53). The level of genetic diversity we uncovered in the vaginal yeast populations suggests a relatively high level of standing genetic variation, particularly since the available comparable WGS studies in other contexts purposefully selected isolates that maximized phenotypical differences (36, 37). Interestingly, the presence of vaginal genetic diversity is consistent with one of the earliest genetic studies, which used DNA fingerprinting to examine diversity at a single time point in up to 14 vaginal isolates from six RVVC populations (54).

All three *C. albicans* RVVC populations were part of a subgroup in the global phylogeny, within clade 1. This subgroup is enriched for vaginal isolates compared to the entire tree and even compared to the other part of clade 1. Compared to different clades, a greater proportion of clade 1 isolates has previously been noted to be significantly associated with superficial infections (55), including in the context of R/VVC (17, 33, 34, 56). There may be something unique to the common ancestor of the clade 1 subgroup that makes them more amenable to colonizing and invading epithelial surfaces in general and hence able to cause vaginal disease (57, 58). Most phenotypes of potential clinical interest have previously been found to vary among isolates within the same clade, precluding clear phenotype × clade associations (59, 60). However, it may be that finer-scale phylogenetic resolution is required to tease apart relationships; if only a subgroup of clade 1 isolates is enriched for a particular phenotype, this might not be seen if all clade 1 isolates are grouped together. Sala et al. recently found that VVC isolates induced greater fungal shedding from epithelial cells and differently stimulated epithelial signaling pathways compared to isolates from healthy women (61). This is the clearest *in vitro* assay able to differentiate VVC and healthy isolates phenotypically, and hence, a strong target for a genome-wide association study to potentially pinpoint the genetic basis of this seemingly important trait. It will be of great interest in the future to determine whether there is a genotypic association between common variants in the subgroup of clade 1 isolates (including isolates from other body sites) and their interaction with vaginal epithelial cells.

Significantly less work has been done to examine *N. glabratus* in the context of R/VVC compared to *C. albicans*, despite the increasing incidence of *N. glabratus* globally as an etiological agent of R/VVC (18, 62). We found only a single vaginal isolate out of 526 total isolates with WGS data on NCBI. This sharply contrasts with *C. albicans*, where 35% of all sequenced isolates in the current phylogeny have been annotated as vaginal origin (38). Surprisingly, vaginal isolates form over 10% of the isolates in the *N. glabratus* MLST database (63). Those isolates are widely distributed among ST groups. This highlights the gap in inclusion of vaginal isolates in *N. glabratus* studies that use WGS.

### Conclusion

We have conducted the most extensive study to date that employed whole-genome sequencing, modern methods for calculating and comparing diversity, and high throughput phenotypic analyses to compare vaginal and rectal isolates from participants with a history of symptomatic RVVC. We find no evidence that rectal isolates are different than vaginal isolates, which is inconsistent with the hypothesis that the GI tract is a source population for vaginal reinfection. We observed a near-identical average nucleotide diversity between our populations and some populations from commensal (*C. albicans*) and BSI (*N. glabratus*) settings. It remains unknown whether these values are low or high relative to other body sites and commensal or infection contexts. This emphasizes the need for further investigation into diversity within fungal microbial communities across various contexts.

## MATERIALS AND METHODS

### Clinical isolates

Seventeen consenting female participants who attended a clinic for individuals with a history of RVVC in Winnipeg, Canada, were sampled at the clinic for possible inclusion in this study. Vaginal and rectal swabs were acquired from all participants; swabs were kept at −4°C or on ice during transport and processed within 5 h of acquisition. Swabs were agitated for ~30 s in 1 mL phosphate-buffered saline (PBS), a dilution series (1, 1:10, and 1:100) was conducted, and 100 µL from each dilution was spread onto SDA and chromogenic *Candida* agar plates. Plates were incubated for 48 h at 30°C. To meet our goals of comparing variation between vaginal and rectal populations and to infer the number of isolates required to accurately measure genotypic diversity, we focused our efforts on the four participants who had high vaginal and rectal populations (>1 × 10^3^ CFU/mL of swab elute). Based on the colony color on chromogenic agar, one was *N. glabratus* (YST6), and three were *C. albicans* (YST7, TVY4, and TVY10). Twelve vaginal and 12 rectal colonies with clear margins were haphazardly isolated from each participant, suspended in 1 mL of 20% glycerol, and kept at −70°C. We collectively refer to these 96 isolates collected from 4 of the 17 participants who met our inclusion criteria for this study as the “THRIVE-yeast” isolates.

### DNA extraction and sequencing

Genomic DNA was extracted from all THRIVE-yeast isolates following a standard phenol-chloroform protocol as previously described (64). DNA quality and concentration were assessed on a Thermo Scientific NanoDrop 2000 and Qubit 2.0 Fluorometer. Genomic DNA was sent to either Microbial Genome Sequencing Center (MIGS; Pittsburgh, PA, USA; YST6 and YST7) or SeqCoast Genomics (Portsmouth, NH, USA; TVY4 and TVY10) for sequencing. At MIGS, sample libraries were prepared using the Illumina DNA Prep kit and sequenced on a NextSeq 2000 using a 300-cycle flow cell kit. The bcl-convert v3.9.3 software was used to assess read quality, demultiplex, and trim adapter sequences. At SeqCoast Genomics, samples were prepared using an Illumina DNA Prep tagmentation kit and sequencing was performed on the Illumina NextSeq2000 platform using a 300-cycle flow cell kit. DRAGEN v3.10.11 was used to assess read quality, demultiplex, and trim adapter sequences.

Three of the 96 isolates (TVY10R13, TVY4R4, and YST7R13) had extremely low coverage (<20×) and were excluded from genomic analysis. The average coverage from the remaining 93 isolates was at least 50×. The fastq files from all THRIVE-yeast have been deposited at the NCBI Sequence Read Archive under BioProject ID PRJNA991137.

In addition to the 93 THRIVE-yeast genomes, we downloaded an additional 182 *C*. *albicans* FASTQ files from the NCBI Sequence Read Archive database (65) from BioProject Accession PRJNA432884 (38) and 526 *N*. *glabratus* FASTQ files from 19 different projects on the SRA database (accessed on 5 February 2022), including 99 *N*. *glabratus* FASTQ files from SRA PRJNA361477 (66) and PRJNA669061 (67) (see Tables S1 and S2).

### Variant calling

The sequence reads were trimmed with Trimmomatic (v0.39) (68) with standard parameters (69). Quality was assessed with FASTQC (http://www.bioinformatics.babraham.ac.uk/projects/fastqc/) and MultiQC (70). *C. albicans* trimmed paired-end reads were mapped using bwa-mem (71) to the SC5314 haplotype A reference genome (A22-s07-m01-r160) downloaded from the Candida Genome Database (CGD) (72). The resulting SAM file was coordinate-sorted and converted to a BAM file using samtools v1.9 (73). *N. glabratus* isolates were mapped to the CBS 138 reference genome (GCA000002545v2) downloaded from the Ensembl Genome Database (74). Alignment quality was assessed with CollectAlignmentSummaryMetrics from Picard v2.26.3 (http://broadinstitute.github.io/picard) and consolidated across all samples with MultiQC (70). All files had a >95% mapping quality. BAM files were further processed with Picard by adding a read group annotation so that samples with the same Bioproject ID had the same read group, removing duplicate PCR amplicons and fixing mate pairs. Base quality scores for the *C. albicans* aligned reads were recalibrated with known single-nucleotide polymorphisms obtained from the Candida Genome Database website (http://www.candidagenome.org/download/gff/C_albicans_SC5314/Assembly22/A22_Jones_PMID_15123810_Polymorphisms.vcf; downloaded on 29 July 2020) (75) using the BaseRecalibrator and ApplyBQSR from the Genome Analysis Toolkit 4.2.4.0. The average coverage for each isolate was estimated using samtools v1.9 (73).

The GATK Best Practices were adapted for variant calling. In sequence, HaplotypeCaller, CombineGVCFs, GenotypeVCFs, VariantFiltration, and SelectVariants (76–78) were used to identify SNPs among all sequenced isolates in diploid and haploid mode for *C. albicans* and *N. glabratus*, respectively. The resulting SNP table was hard filtered using the suggested parameters and to match (38) (QualByDepth < 2.0, FisherStrand > 60.0, root mean square mapping quality < 30.0, MappingQualityRankSumTest < −12.5, and ReadPosRankSumTest < −8.0). We excluded variants that were called in known repetitive regions of the genome, as these are likely to reflect sequencing misalignments rather than true variants, i.e., the subtelomeric regions (15 kb from the start and end of each chromosome), the centromeres, and the major repeat sequence regions (Table S3, start and stop positions from candidagenome.org).

### Phylogeny construction

Phylogenetic trees were constructed for *C. albicans* and *N. glabratus*. For each species, the multi-sample VCF file consisting of genomic SNPs was converted to a FASTA alignment using vcf2phylip.py v2.8 (79). For heterozygous SNPs in *C. albicans*, the consensus sequence is preferentially made based on the reference (haplotype A) base. Ambiguous bases are written following IUPAC nucleotide ambiguity codes in the matrix. The FASTA alignment was parsed in FastTree (2.1.11) (80) in the double precision mode to construct an approximate maximum-likelihood phylogenetic tree using the general time reversible model and the -gamma option to rescale the branch lengths. The phylogeny was visualized and annotated with the Interactive Tree Of Life (iTOL, v5) (81). Isolates from *C. albicans* clade 13, i.e., *C. africana* (39, 40, 82), were used to root the phylogeny. The *N. glabratus* phylogeny was rooted at the midpoint. Following standard practice for *N. glabratus*, we used MLST analysis (66, 67) to identify and name the YST6 ST group. An *in silico* MLST analysis from the fastq data was done using stringMLST (83) using the predefined MLST *N. glabratus* allele library (*FKS*, *LEU2*, *NMT1*, *TRP1*, *UGP1*, and *URA3*) from the PubMLST database (84).

To phylogenetically compare the vaginal and rectal isolates within each participant, we constructed a maximum likelihood phylogeny for each with RAxML v8.2.12 following standard practice using the GTR + G model with 20 mL inferences on the alignment, inferring bootstrap replicate trees, applying MRE-based bootstrapping test, and drawing support values using transfer bootstrap expectation on the best-scoring tree (41).

### Diversity analysis of isolates from each participant

RTG tools with the vcfsplit option (85) were used to extract VCF files for each isolate from the multi-sample VCF file. The VCF files were converted to bed files using “bcftools query -f.” A custom R script was used to conduct a pairwise comparison of the isolates from a site to determine differences in SNP positions (“05a_SNP_difference.R” and “05b_SNP_difference_plotStats.R”). An ANOVA test was performed to compare pairwise SNP differences in rectal and vaginal isolates from each participant.

A principal component analysis on windows of genomic regions that differed among isolates from the same participant was conducted. The “templated script” from the R package lostruct (local PCA/population structure, v.0.0.0.9 [86]) was run with parameters -t: bp, -s: 5000, -npc: 2, and -m: 2. To check for possible segregation of vaginal and rectal isolates, plots were generated based on the most extreme heterogeneous genomic windows across the entire genome and visually examined.

### Relationship between average nucleotide diversity (π) and number of samples

The potential influence of the sample size was assessed using the average pairwise diversity differences between all possible isolate pair estimates from Pixy (v1.2.6.beta1 [87]). Briefly, variants were called using GenotypeGVCFs with --all-sites option activated. Vcftools (v0.1.16) (88) was used to filter the variants (with --max-meanDP 500, --min-meanDP 20, and --max-missing 0.8). Indels and mitochondrial DNA were excluded (--remove-indels and --not-chr). For each sample size, we randomly selected *n* vaginal isolates 50 times without replacement and calculated π for each group.

### *In silico* mating-type locus detection

To determine the mating type-like locus in the *C. albicans* isolates (YST7, TVY4, and TVY10), the reads were aligned to both haplotypes, and consensus sequences of the MAT locus on chromosome 5 (MATa1 and MATa2 for hapA and MATα1 and MATα2) were extracted. A BLAST search was then conducted to confirm the locus. Similarly, the mating type-like locus of *N. glabratus* (YST6) isolates was determined by determining the consensus sequence for MTL1 (MTLalpha1 and MTLalpha2) and MTL3 on chromosome B, and MTL2 on chromosome E, and confirming the MTL by a BLAST search.

### Loss of heterozygosity and copy number analyses

LOH and CNV analyses were conducted using the web-based yeast analysis pipeline (Y_MAP_) (42) *N. glabratus* isolates were compared to the CBS138 reference genome (CGD: s05-m01-r09). *C. albicans* isolate was analyzed against the SC5314 A22-s02-m09-r10 reference genome. Correction was enabled for GC-content bias and chromosome-end bias. The genomic elements within observed CNV regions were identified using the “gene/sequence resources” section of the CGD (http://candidagenome.org/).

### Growth rate assay

Two separate growth rate assays were conducted to measure growth in RPMI (10.4% [wt/vol] RPMI powder, 1.5% [wt/vol] dextrose, 1.73% [wt/vol] 3-[N-morpholino] propanesulfonic acid, adjusted to pH seven with NaOH tablets) and VSM (following reference 38: 1.16% [vol/vol] 5 mM NaCl, 3.6% [vol/vol] 0.5 M KOH, 0.0128% [vol/vol] 99% glycerol, 20% [vol/vol] 0.01 M Ca[OH]_2_, 1.34% [vol/vol] 0.5 M urea, 6.6% [vol/vol] 0.5 M glucose, 0.67% [wt/vol] solid YNB, 0.85% [vol/vol] 2 M acetic acid, and 0.192% [vol/vol] lactic acid, adjusted to pH 4.2 with NaOH tablets). For each, 5 µL of frozen glycerol stock from all THRIVE-yeast isolates was inoculated in duplicate into 500 µL of RPMI or VSM and incubated for 48 h at 37°C with agitation at 250 rpm. Cultures were standardized to an optical density (OD) of 0.01 A600 in RPMI or VSM, and 200 µL was transferred into a 96-well round-bottom plate and sealed with a Breathe-Easier sealing membrane (Electron Microscopy Sciences, PA, United States). OD_600_ readings were taken by the Epoch plate reader (Biotek) every 15 minutes, with continuous shaking at 37°C for 48 h. From each well, the maximal growth rate was calculated as the spline with the highest slope using a custom R script written by Dr. Richard Fitzjohn (https://github.com/MicroStatsLab/Microstats/blob/main/R/growthRates.R). The average growth rate between two technical replicates for each isolate in each growth medium was used for visualization and statistical analysis. Statistical outliers were determined through Rosner’s test of outliers available through the rosterTest function in the EnvStats R package (89). For each population, we started with a *k* value of one (i.e., testing for a single outlier). If that was significant, we increased *k* by one until no additional outliers were identified.

### Drug resistance and tolerance

Disk diffusion assays were performed to quantify variation among isolates in resistance and tolerance. A pilot experiment was done on 24 isolates from YST7 in five different drugs (FLC, clotrimazole [CLT], miconazole [MCZ], NYT, and BA). Subsequently, disk diffusion assays were conducted on all isolates to fluconazole and boric acid at pH 4.2. We chose to focus our efforts on fluconazole and boric acid, as these are drugs in different classes that are both treatment options for induction and maintenance therapy of recurrent VVC, and boric acid is used in the treatment of non-albicans VVC (16). Except for the pH adjustment, the protocol outlined in the NCCLS M44 guidelines for antifungal disk diffusion susceptibility testing for fluconazole was followed (90) and adapted for boric acid as previously described (44). The entire experiment was conducted twice for each isolate × drug, with two technical replicates for each of the two biological replicates. Previous work demonstrated consistent drug resistance values at 24 h and 48 h, with drug tolerance apparent at 48 h. Photographs were taken at 48 h images and processed in ImageJ as previously described (44), then run through the diskImageR package (47) for drug resistance (RAD_20_) and tolerance (FOG_20_) quantification. Briefly, diskImageR calculates resistance as RAD_20_, as the radius of the zone of inhibition where growth is reduced by 20% relative to growth on the margins of the plate where there is no drug, and tolerance as FoG_20_, the fraction of realized growth between RAD_20_ and the disk.

A Welch two-sample *t*-test that did not assume equal variance was used for each participant × drug combination to compare vaginal and rectal isolates. Statistical outliers were determined through Rosner’s test of outliers for growth rates. All statistical analysis was done at a type I error rate of 0.05.

### Invasive growth assay

To examine invasive growth, we revised methods from reference 91. Freezer stock from THRIVE-yeast isolates was streaked onto 20 mL yeast peptone dextrose (YPD) plates (2% [wt/vol] peptone, 2% [wt/vol] yeast extract, 1.8% [wt/vol] agar, 1% [wt/vol] glucose, 0.00016% [wt/vol] adenine sulfate, 0.00008% [wt/vol] uridine, and 0.1% [vol/vol] of chloramphenicol and ampicillin) and grown for 72 h at room temperature. A single colony was then randomly chosen from each isolate and inoculated into 200 µL YPD. If no single colonies were available, a similar amount of culture from the colony lawn was used. Cultures were standardized to OD_600_ 0.01 in 1 mL of liquid YPD media, and then 2 µL of standardized culture was spotted onto the surface of a 20 mL solid YPD plate in a hexagonal pattern for a total of 7 spots per plate (i.e., spotted at each vertex and in the center). Plates were incubated for 96 h at 37°C. The surface growth was washed off using distilled water, and a photograph was taken in a dark room on a lightbox. Two biological replicates were performed for each isolate.

The qualitative amount of invasive growth for each isolate was determined by visual examination of the post-wash photographs. To develop a five-point scale, two different people independently went through the post-wash pictures from YST6 and YST7 and selected two to six representative pictures that fit into five levels of invasive growth (scored as 1–5). The independent selections were then compared, and one image from each person was chosen as the most representative for each level of the scale. Using these as a reference, each isolate was then categorized into the five levels of the scale: 0 (no growth/pipette tip indent), 0.25 (pinprick growth), 0.5 (circular growth evident), 0.75 (circular growth with pinprick), and 1 (dense growth throughout). The maximum score between the two bio-replicates of each isolate was used for statistical analysis, though the same statistical conclusions were obtained if the mean score was used instead. For each participant, a Wilcoxon rank sum test was used to compare vaginal and rectal isolates.

## Data Availability

FASTQ files generated for this project have been deposited at the National Center for Biotechnology Information (NCBI) Sequence Read Archive under BioProject ID PRJNA991137. All phenotypic data and code required to reproduce figures and statistical analyses are available at https://github.com/acgerstein/THRIVE_yeast-VR. Large files (e.g., BAM and VCF) are available upon request.

## SUPPLEMENTAL MATERIAL

Supplemental Figures msystems.00157-25-s0001.pdf Figures S1 to S4.

Table S1 msystems.00157-25-s0002.xlsx *N. glabratus* isolates used.

Table S2 msystems.00157-25-s0003.xlsx *Candida albicans* isolates used.

Table S3 msystems.00157-25-s0004.xlsx *N. glabratus* regions excluded in the genomic analyses.

Table S4 msystems.00157-25-s0005.xlsx Statistical analyses of the quantified phenotypes.

ASM does not own the copyrights to Supplemental Material that may be linked to, or accessed through, an article. The authors have granted ASM a non-exclusive, world-wide license to publish the Supplemental Material files. Please contact the corresponding author directly for reuse.

## References

[1] Benedict K, Singleton AL, Jackson BR, Molinari NAM. 2022. Survey of incidence, lifetime prevalence, and treatment of self-reported vulvovaginal candidiasis, United States, 2020. BMC Womens Health 22:147. doi:10.1186/s12905-022-01741-x 35538480 PMC9092842

[2] Yano J, Sobel JD, Nyirjesy P, Sobel R, Williams VL, Yu Q, Noverr MC, Fidel PL Jr. 2019. Current patient perspectives of vulvovaginal candidiasis: incidence, symptoms, management and post-treatment outcomes. BMC Womens Health 19:48. doi:10.1186/s12905-019-0748-8 30925872 PMC6441174

[3] Rathod SD, Buffler PA. 2014. Highly-cited estimates of the cumulative incidence and recurrence of vulvovaginal candidiasis are inadequately documented. BMC Womens Health 14:43. doi:10.1186/1472-6874-14-43 24612727 PMC3975582

[4] Denning DW, Kneale M, Sobel JD, Rautemaa-Richardson R. 2018. Global burden of recurrent vulvovaginal candidiasis: a systematic review. Lancet Infect Dis 18:e339–e347. doi:10.1016/S1473-3099(18)30103-8 30078662

[5] Foxman B, Barlow R, D’Arcy H, Gillespie B, Sobel JD. 2000. Candida vaginitis: self-reported incidence and associated costs. Sex Transm Dis 27:230–235. doi:10.1097/00007435-200004000-00009 10782746

[6] Benyas D, Sobel JD. 2022. Mixed vaginitis due to bacterial vaginosis and candidiasis. J Low Genit Tract Dis 26:68–70. doi:10.1097/LGT.0000000000000641 34840242

[7] Kazmerski TM, Sawicki GS, Miller E, Jones KA, Abebe KZ, Tuchman LK, Ladores S, Rubenstein RC, Sagel SD, Weiner DJ, Pilewski JM, Orenstein DM, Borrero S. 2018. Sexual and reproductive health behaviors and experiences reported by young women with cystic fibrosis. J Cyst Fibros 17:57–63. doi:10.1016/j.jcf.2017.07.017 28774749

[8] Fischer G, Bradford J. 2011. Vulvovaginal candidiasis in postmenopausal women: the role of hormone replacement therapy. J Low Genit Tract Dis 15:263–267. doi:10.1097/LGT.0b013e3182241f1a 21959570

[9] Babula O, Lazdane G, Kroica J, Ledger WJ, Witkin SS. 2003. Relation between recurrent vulvovaginal candidiasis, vaginal concentrations of mannose-binding lectin, and a mannose-binding lectin gene polymorphism in Latvian women. Clin Infect Dis 37:733–737. doi:10.1086/377234 12942410

[10] Wojitani M, de Aguiar LM, Baracat EC, Linhares IM. 2012. Association between mannose-binding lectin and interleukin-1 receptor antagonist gene polymorphisms and recurrent vulvovaginal candidiasis. Arch Gynecol Obstet 285:149–153. doi:10.1007/s00404-011-1920-z 21655939

[11] Rosentul DC, Delsing CE, Jaeger M, Plantinga TS, Oosting M, Costantini I, Venselaar H, Joosten LAB, van der Meer JWM, Dupont B, Kullberg B-J, Sobel JD, Netea MG. 2014. Gene polymorphisms in pattern recognition receptors and susceptibility to idiopathic recurrent vulvovaginal candidiasis. Front Microbiol 5:483. doi:10.3389/fmicb.2014.00483 25295030 PMC4172055

[12] Sobel JD. 2003. Management of patients with recurrent vulvovaginal candidiasis. Drugs (Abingdon Engl) 63:1059–1066. doi:10.2165/00003495-200363110-00002 12749733

[13] Zhang J-Y, Liu J-H, Liu F-D, Xia Y-H, Wang J, Liu X, Zhang Z-Q, Zhu N. 2014. Vulvovaginal candidiasis: species distribution, fluconazole resistance and drug efflux pump gene overexpression. Mycoses 57:584–591. doi:10.1111/myc.12204 24962255

[14] Shi X-Y, Yang Y-P, Zhang Y, Li W, Wang J-D, Huang W-M, Fan Y-M. 2015. Molecular identification and antifungal susceptibility of 186 Candida isolates from vulvovaginal candidiasis in southern China. J Med Microbiol 64:390–393. doi:10.1099/jmm.0.000024 25596116

[15] Guzel AB, Ilkit M, Akar T, Burgut R, Demir SC. 2011. Evaluation of risk factors in patients with vulvovaginal candidiasis and the value of chromID Candida agar versus CHROMagar Candida for recovery and presumptive identification of vaginal yeast species. Med Mycol 49:16–25. doi:10.3109/13693786.2010.497972 20608776

[16] van Schalkwyk J, Yudin MH, INFECTIOUS DISEASE COMMITTEE. 2015. Vulvovaginitis: screening for and management of trichomoniasis, vulvovaginal candidiasis, and bacterial vaginosis. J Obstet Gynaecol Can 37:266–274. doi:10.1016/S1701-2163(15)30316-9 26001874

[17] Song N, Kan S, Pang Q, Mei H, Zheng H, Li D, Cui F, Lv G, An R, Li P, Xiong Z, Fan S, Zhang M, Chen Y, Qiao Q, Liang X, Cui M, Li D, Liao Q, Li X, Liu W. 2022. A prospective study on vulvovaginal candidiasis: multicentre molecular epidemiology of pathogenic yeasts in China. J Eur Acad Dermatol Venereol 36:566–572. doi:10.1111/jdv.17874 34908189

[18] Kennedy MA, Sobel JD. 2010. Vulvovaginal candidiasis caused by non-albicans Candida species: new insights. Curr Infect Dis Rep 12:465–470. doi:10.1007/s11908-010-0137-9 21308556

[19] Parazzini F, Di Cintio E, Chiantera V, Guaschino S. 2000. Determinants of different Candida species infections of the genital tract in women. Eur J Obstet Gynecol Reprod Biol 93:141–145. doi:10.1016/s0301-2115(00)00289-x 11074134

[20] Richter SS, Galask RP, Messer SA, Hollis RJ, Diekema DJ, Pfaller MA. 2005. Antifungal susceptibilities of Candida species causing vulvovaginitis and epidemiology of recurrent cases. J Clin Microbiol 43:2155–2162. doi:10.1128/JCM.43.5.2155-2162.2005 15872235 PMC1153777

[21] Borman AM, Johnson EM. 2021. Name changes for fungi of medical importance, 2018 to 2019. J Clin Microbiol 59:e01811-20. doi:10.1128/JCM.01811-20 33028600 PMC8111128

[22] Kidd SE, Abdolrasouli A, Hagen F. 2023. Fungal nomenclature: managing change is the name of the game. Open Forum Infect Dis 10:ofac559. doi:10.1093/ofid/ofac559 36632423 PMC9825814

[23] Pfaller MA, Rhomberg PR, Messer SA, Jones RN, Castanheira M. 2015. Isavuconazole, micafungin, and 8 comparator antifungal agents’ susceptibility profiles for common and uncommon opportunistic fungi collected in 2013: temporal analysis of antifungal drug resistance using CLSI species-specific clinical breakpoints and proposed epidemiological cutoff values. Diagn Microbiol Infect Dis 82:303–313. doi:10.1016/j.diagmicrobio.2015.04.008 25986029

[24] Drell T, Lillsaar T, Tummeleht L, Simm J, Aaspõllu A, Väin E, Saarma I, Salumets A, Donders GGG, Metsis M. 2013. Characterization of the vaginal micro- and mycobiome in asymptomatic reproductive-age Estonian women. PLoS One 8:e54379. doi:10.1371/journal.pone.0054379 23372716 PMC3553157

[25] Tasić S, Tasić N, Tasić A, Mitrović S. 2002. Recurrent genital candidiasis ofwomen: consequence of reinfection or replace. J Med Biol 9:214–222. doi:10.1056/NEJM198612043152305

[26] Milne JD, Warnock DW. 1979. Effect of simultaneous oral and vaginal treatment on the rate of cure and relapse in vaginal candidosis. Br J Vener Dis 55:362–365. doi:10.1136/sti.55.5.362 389354 PMC1045682

[27] El-Din SS, Reynolds MT, Ashbee HR, Barton RC, Evans EG. 2001. An investigation into the pathogenesis of vulvo-vaginal candidosis. Sex Transm Infect 77:179–183. doi:10.1136/sti.77.3.179 11402224 PMC1744307

[28] Fong IW. 1994. The rectal carriage of yeast in patients with vaginal candidiasis. Clin Invest Med 17:426–431. 7867247

[29] Sobel JD. 1986. Recurrent vulvovaginal candidiasis. N Engl J Med 315:1455–1458. doi:10.1056/NEJM198612043152305 3537785

[30] Mårdh P-A, Novikova N, Stukalova E. 2003. Colonisation of extragenital sites by Candida in women with recurrent vulvovaginal candidosis. BJOG 110:934–937. doi:10.1111/j.1471-0528.2003.01445.x 14550364

[31] Spinillo A, Nicola S, Colonna L, Marangoni E, Cavanna C, Michelone G. 1994. Frequency and significance of drug resistance in vulvovaginal candidiasis. Gynecol Obstet Invest 38:130–133. doi:10.1159/000292465 7959341

[32] O’Connor MI, Sobel JD. 1986. Epidemiology of recurrent vulvovaginal candidiasis: identification and strain differentiation of Candida albicans. J Infect Dis 154:358–363. doi:10.1093/infdis/154.2.358 3522761

[33] Tian J-Y, Yang Y-G, Chen S, Teng Y, Li X-Z. 2021. Genetic diversity and molecular epidemiology of Candida albicans from vulvovaginal candidiasis patients. Infect Genet Evol 92:104893. doi:10.1016/j.meegid.2021.104893 33964472

[34] Zhu Y, Fang C, Shi Y, Shan Y, Liu X, Liang Y, Huang L, Liu X, Liu C, Zhao Y, Fan S, Zhang X. 2022. Candida albicans multilocus sequence typing clade I contributes to the clinical phenotype of vulvovaginal candidiasis patients. Front Med 9:837536. doi:10.3389/fmed.2022.837536 PMC9010739 35433756

[35] Sitterlé E, Maufrais C, Sertour N, Palayret M, d’Enfert C, Bougnoux M-E. 2019. Within-host genomic diversity of Candida albicans in healthy carriers. Sci Rep 9:2563. doi:10.1038/s41598-019-38768-4 30796326 PMC6385308

[36] Anderson FM, Visser ND, Amses KR, Hodgins-Davis A, Weber AM, Metzner KM, McFadden MJ, Mills RE, O’Meara MJ, James TY, O’Meara TR. 2023. Candida albicans selection for human commensalism results in substantial within-host diversity without decreasing fitness for invasive disease. PLoS Biol 21:e3001822. doi:10.1371/journal.pbio.3001822 37205709 PMC10234564

[37] Badrane H, Cheng S, Dupont CL, Hao B, Driscoll E, Morder K, Liu G, Newbrough A, Fleres G, Kaul D, Espinoza JL, Clancy CJ, Nguyen MH. 2023. Genotypic diversity and unrecognized antifungal resistance among populations of Candida glabrata from positive blood cultures. Nat Commun 14:5918. doi:10.1038/s41467-023-41509-x 37739935 PMC10516878

[38] Ropars J, Maufrais C, Diogo D, Marcet-Houben M, Perin A, Sertour N, Mosca K, Permal E, Laval G, Bouchier C, et al.. 2018. Gene flow contributes to diversification of the major fungal pathogen Candida albicans. Nat Commun 9:2253. doi:10.1038/s41467-018-04787-4 29884848 PMC5993739

[39] Romeo O, Tietz H-J, Criseo G. 2013. Candida africana: is it a fungal pathogen? Curr Fungal Infect Rep 7:192–197. doi:10.1007/s12281-013-0142-1

[40] Mixão V, Saus E, Boekhout T, Gabaldón T. 2021. Extreme diversification driven by parallel events of massive loss of heterozygosity in the hybrid lineage of Candida albicans. Genetics 217:iyaa004. doi:10.1093/genetics/iyaa004 33724404 PMC8045679

[41] Stamatakis A. 2014. RAxML version 8: a tool for phylogenetic analysis and post-analysis of large phylogenies. Bioinformatics 30:1312–1313. doi:10.1093/bioinformatics/btu033 24451623 PMC3998144

[42] Abbey DA, Funt J, Lurie-Weinberger MN, Thompson DA, Regev A, Myers CL, Berman J. 2014. YMAP: a pipeline for visualization of copy number variation and loss of heterozygosity in eukaryotic pathogens. Genome Med 6:100. doi:10.1186/s13073-014-0100-8 25505934 PMC4263066

[43] Pemberton TJ. 2006. Identification and comparative analysis of sixteen fungal peptidyl-prolyl cis/trans isomerase repertoires. BMC Genomics 7:244. doi:10.1186/1471-2164-7-244 16995943 PMC1618848

[44] Salama OE, Gerstein AC. 2022. Differential response of Candida species morphologies and isolates to fluconazole and boric acid. Antimicrob Agents Chemother 66:e02406-21. doi:10.1128/aac.02406-21 35446135 PMC9112882

[45] Bhattacharjee P. 2016. Epidemiology and antifungal susceptibility of Candida species in a tertiary care hospital, Kolkata, India. Curr Med Mycol 2:20–27. doi:10.18869/acadpub.cmm.2.2.5 PMC5490301 28681016

[46] Venkateswarlu K, Taylor M, Manning NJ, Rinaldi MG, Kelly SL. 1997. Fluconazole tolerance in clinical isolates of Cryptococcus neoformans. Antimicrob Agents Chemother 41:748–751. doi:10.1128/AAC.41.4.748 9087482 PMC163787

[47] Gerstein AC, Rosenberg A, Hecht I, Berman J. 2016. diskImageR: quantification of resistance and tolerance to antimicrobial drugs using disk diffusion assays. Microbiology (Reading) 162:1059–1068. doi:10.1099/mic.0.000295 27126388 PMC5756480

[48] Araújo Paulo de Medeiros M, Vieira de Melo AP, Gonçalves SS, Milan EP, Chaves GM. 2014. Genetic relatedness among vaginal and anal isolates of Candida albicans from women with vulvovaginal candidiasis in north-east Brazil. J Med Microbiol 63:1436–1445. doi:10.1099/jmm.0.076604-0 25187602

[49] Sampaio P, Gusmão L, Alves C, Pina-Vaz C, Amorim A, Pais C. 2003. Highly polymorphic microsatellite for identification of Candida albicans strains. J Clin Microbiol 41:552–557. doi:10.1128/JCM.41.2.552-557.2003 12574245 PMC149659

[50] Shi W, Mei X, Gao F, Huo K, Shen L, Qin H, Wu Z, Zheng J. 2007. Analysis of genital Candida albicans infection by rapid microsatellite markers genotyping. Chin Med J 120:975–980. doi:10.1097/00029330-200706010-00007 17624265

[51] Araújo Paulo de Medeiros M, Vieira de Melo AP, Maia de Sousa AM, Silva-Rocha WP, Pipolo Milan E, Maranhão Chaves G. 2017. Characterization of virulence factors of vaginal and anal isolates of Candida albicans sequentially obtained from patients with vulvovaginal candidiasis in north-east Brazil. J Mycol Med 27:567–572. doi:10.1016/j.mycmed.2017.06.002 28844452

[52] Lemberg C, Martinez de San Vicente K, Fróis-Martins R, Altmeier S, Tran VDT, Mertens S, Amorim-Vaz S, Rai LS, d’Enfert C, Pagni M, Sanglard D, LeibundGut-Landmann S. 2022. Candida albicans commensalism in the oral mucosa is favoured by limited virulence and metabolic adaptation. PLoS Pathog 18:e1010012. doi:10.1371/journal.ppat.1010012 35404986 PMC9041809

[53] Qu W-M, Liang N, Wu Z-K, Zhao Y-G, Chu D. 2020. Minimum sample sizes for invasion genomics: empirical investigation in an invasive whitefly. Ecol Evol 10:38–49. doi:10.1002/ece3.5677 31988715 PMC6972819

[54] Lockhart SR, Fritch JJ, Meier AS, Schröppel K, Srikantha T, Galask R, Soll DR. 1995. Colonizing populations of Candida albicans are clonal in origin but undergo microevolution through C1 fragment reorganization as demonstrated by DNA fingerprinting and C1 sequencing. J Clin Microbiol 33:1501–1509. doi:10.1128/jcm.33.6.1501-1509.1995 7650175 PMC228204

[55] Odds FC, Bougnoux M-E, Shaw DJ, Bain JM, Davidson AD, Diogo D, Jacobsen MD, Lecomte M, Li S-Y, Tavanti A, Maiden MCJ, Gow NAR, d’Enfert C. 2007. Molecular phylogenetics of Candida albicans. Eukaryot Cell 6:1041–1052. doi:10.1128/EC.00041-07 17416899 PMC1951527

[56] Ge S-H, Xie J, Xu J, Li J, Li D-M, Zong L-L, Zheng Y-C, Bai F-Y. 2012. Prevalence of specific and phylogenetically closely related genotypes in the population of Candida albicans associated with genital candidiasis in China. Fungal Genet Biol 49:86–93. doi:10.1016/j.fgb.2011.10.006 22079546

[57] Schmid J, Herd S, Hunter PR, Cannon RD, Yasin MSM, Samad S, Carr M, Parr D, McKinney W, Schousboe M, Harris B, Ikram R, Harris M, Restrepo A, Hoyos G, Singh KP. 1999. Evidence for a general-purpose genotype in Candida albicans, highly prevalent in multiple geographical regions, patient types and types of infection. Microbiology (Reading) 145:2405–2413. doi:10.1099/00221287-145-9-2405 10517593

[58] Giblin L, Edelmann A, Zhang N, von Maltzahn NB, Cleland SB, Sullivan PA, Schmid J. 2001. A DNA polymorphism specific to Candida albicans strains exceptionally successful as human pathogens. Gene 272:157–164. doi:10.1016/s0378-1119(01)00548-0 11470521

[59] Cravener MV, Do E, May G, Zarnowski R, Andes DR, McManus CJ, Mitchell AP. 2023. Reinforcement amid genetic diversity in the Candida albicans biofilm regulatory network. PLoS Pathog 19:e1011109. doi:10.1371/journal.ppat.1011109 36696432 PMC9901766

[60] MacCallum DM, Castillo L, Nather K, Munro CA, Brown AJP, Gow NAR, Odds FC. 2009. Property differences among the four major Candida albicans strain clades. Eukaryot Cell 8:373–387. doi:10.1128/EC.00387-08 19151328 PMC2653250

[61] Sala A, Ardizzoni A, Spaggiari L, Vaidya N, van der Schaaf J, Rizzato C, Cermelli C, Mogavero S, Krüger T, Himmel M, Kniemeyer O, Brakhage AA, King BL, Lupetti A, Comar M, de Seta F, Tavanti A, Blasi E, Wheeler RT, Pericolini E. 2023. A new phenotype in Candida-epithelial cell interaction distinguishes colonization- versus vulvovaginal candidiasis-associated strains. mBio 14:e00107-23. doi:10.1128/mbio.00107-23 36856418 PMC10128025

[62] Makanjuola O, Bongomin F, Fayemiwo SA. 2018. An update on the roles of non-albicans Candida species in vulvovaginitis. J Fungi 4:121. doi:10.3390/jof4040121 PMC6309050 30384449

[63] Dodgson AR, Pujol C, Denning DW, Soll DR, Fox AJ. 2003. Multilocus sequence typing of Candida glabrata reveals geographically enriched clades. J Clin Microbiol 41:5709–5717. doi:10.1128/JCM.41.12.5709-5717.2003 14662965 PMC309006

[64] Kukurudz RJ, Chapel M, Wonitowy Q, Adamu Bukari A-R, Sidney B, Sierhuis R, Gerstein AC. 2022. Acquisition of cross-azole tolerance and aneuploidy in Candida albicans strains evolved to posaconazole. G3 (Bethesda) 12:jkac156. doi:10.1093/g3journal/jkac156 35881695 PMC9434289

[65] Sayers EW, Bolton EE, Brister JR, Canese K, Chan J, Comeau DC, Connor R, Funk K, Kelly C, Kim S, Madej T, Marchler-Bauer A, Lanczycki C, Lathrop S, Lu Z, Thibaud-Nissen F, Murphy T, Phan L, Skripchenko Y, Tse T, Wang J, Williams R, Trawick BW, Pruitt KD, Sherry ST. 2022. Database resources of the national center for biotechnology information. Nucleic Acids Res 50:D20–D26. doi:10.1093/nar/gkab1112 34850941 PMC8728269

[66] Carreté L, Ksiezopolska E, Pegueroles C, Gómez-Molero E, Saus E, Iraola-Guzmán S, Loska D, Bader O, Fairhead C, Gabaldón T. 2018. Patterns of genomic variation in the opportunistic pathogen Candida glabrata suggest the existence of mating and a secondary association with humans. Curr Biol 28:15–27. doi:10.1016/j.cub.2017.11.027 29249661 PMC5772174

[67] Helmstetter N, Chybowska AD, Delaney C, Da Silva Dantas A, Gifford H, Wacker T, Munro C, Warris A, Jones B, Cuomo CA, Wilson D, Ramage G, Farrer RA. 2022. Population genetics and microevolution of clinical Candida glabrata reveals recombinant sequence types and hyper-variation within mitochondrial genomes, virulence genes, and drug targets. Genetics 221:iyac031. doi:10.1093/genetics/iyac031 35199143 PMC9071574

[68] Bolger AM, Lohse M, Usadel B. 2014. Trimmomatic: a flexible trimmer for Illumina sequence data. Bioinformatics 30:2114–2120. doi:10.1093/bioinformatics/btu170 24695404 PMC4103590

[69] Todd RT, Wikoff TD, Forche A, Selmecki A. 2019. Genome plasticity in Candida albicans is driven by long repeat sequences. Elife 8:e45954. doi:10.7554/eLife.45954 31172944 PMC6591007

[70] Ewels P, Magnusson M, Lundin S, Käller M. 2016. MultiQC: summarize analysis results for multiple tools and samples in a single report. Bioinformatics 32:3047–3048. doi:10.1093/bioinformatics/btw354 27312411 PMC5039924

[71] Li H. 2013. Aligning sequence reads, clone sequences and assembly contigs with BWA-MEM. arXiv. doi:10.48550/arXiv.1303.3997

[72] Skrzypek MS, Binkley J, Binkley G, Miyasato SR, Simison M, Sherlock G. 2017. The Candida Genome Database (CGD): incorporation of Assembly 22, systematic identifiers and visualization of high throughput sequencing data. Nucleic Acids Res 45:D592–D596. doi:10.1093/nar/gkw924 27738138 PMC5210628

[73] Li H, Handsaker B, Wysoker A, Fennell T, Ruan J, Homer N, Marth G, Abecasis G, Durbin R, Genome Project Data Processing Subgroup. 2009. The sequence alignment/map format and SAMtools. Bioinformatics 25:2078–2079. doi:10.1093/bioinformatics/btp352 19505943 PMC2723002

[74] Yates AD, Allen J, Amode RM, Azov AG, Barba M, Becerra A, Bhai J, Campbell LI, Carbajo Martinez M, Chakiachvili M, et al.. 2022. Ensembl Genomes 2022: an expanding genome resource for non-vertebrates. Nucleic Acids Res 50:D996–D1003. doi:10.1093/nar/gkab1007 34791415 PMC8728113

[75] Jones T, Federspiel NA, Chibana H, Dungan J, Kalman S, Magee BB, Newport G, Thorstenson YR, Agabian N, Magee PT, Davis RW, Scherer S. 2004. The diploid genome sequence of Candida albicans. Proc Natl Acad Sci USA 101:7329–7334. doi:10.1073/pnas.0401648101 15123810 PMC409918

[76] DePristo MA, Banks E, Poplin R, Garimella KV, Maguire JR, Hartl C, Philippakis AA, del Angel G, Rivas MA, Hanna M, McKenna A, Fennell TJ, Kernytsky AM, Sivachenko AY, Cibulskis K, Gabriel SB, Altshuler D, Daly MJ. 2011. A framework for variation discovery and genotyping using next-generation DNA sequencing data. Nat Genet 43:491–498. doi:10.1038/ng.806 21478889 PMC3083463

[77] Van der Auwera GA, Carneiro MO, Hartl C, Poplin R, Del Angel G, Levy-Moonshine A, Jordan T, Shakir K, Roazen D, Thibault J, Banks E, Garimella KV, Altshuler D, Gabriel S, DePristo MA. 2013. From FastQ data to high confidence variant calls: the Genome Analysis Toolkit best practices pipeline. Curr Protoc Bioinformatics 43:11. doi:10.1002/0471250953.bi1110s43 PMC4243306 25431634

[78] Poplin R, Ruano-Rubio V, DePristo MA, Fennell TJ, Carneiro MO, Auwera GA, Kling DE, Gauthier LD, Levy-Moonshine A, Roazen D, Shakir K, Thibault J, Chandran S, Whelan C, Lek M, Gabriel S, Daly MJ, Neale B, MacArthur DG, Banks E. 2018. Scaling accurate genetic variant discovery to tens of thousands of samples. bioRxiv. doi:10.1101/201178

[79] Ortiz EM. 2019. vcf2phylip v2.0: convert a VCF matrix into several matrix formats for phylogenetic analysis. Available from: https://zenodo.org/record/2540861

[80] Price MN, Dehal PS, Arkin AP. 2010. FastTree 2 – approximately maximum-likelihood trees for large alignments. PLoS One 5:e9490. doi:10.1371/journal.pone.0009490 20224823 PMC2835736

[81] Letunic I, Bork P. 2021. Interactive Tree Of Life (iTOL) v5: an online tool for phylogenetic tree display and annotation. Nucleic Acids Res 49:W293–W296. doi:10.1093/nar/gkab301 33885785 PMC8265157

[82] Mixão V, Gabaldón T. 2020. Genomic evidence for a hybrid origin of the yeast opportunistic pathogen Candida albicans. BMC Biol 18:48. doi:10.1186/s12915-020-00776-6 32375762 PMC7204223

[83] Gupta A, Jordan IK, Rishishwar L. 2017. stringMLST: a fast k-mer based tool for multilocus sequence typing. Bioinformatics 33:119–121. doi:10.1093/bioinformatics/btw586 27605103

[84] Jolley KA, Bray JE, Maiden MCJ. 2018. Open-access bacterial population genomics: BIGSdb software, the PubMLST.org website and their applications. Wellcome Open Res 3:124. doi:10.12688/wellcomeopenres.14826.1 30345391 PMC6192448

[85] Cleary JG, Braithwaite R, Gaastra K, Hilbush BS, Inglis S, Irvine SA, Jackson A, Littin R, Rathod M, Ware D, Zook JM, Trigg L, Vega FM. 2015. Comparing variant call files for performance benchmarking of next-generation sequencing variant calling pipelines. bioRxiv. doi:10.1101/023754

[86] Li H, Ralph P. 2019. Local PCA shows how the effect of population structure differs along the genome. Genetics 211:289–304. doi:10.1534/genetics.118.301747 30459280 PMC6325702

[87] Korunes KL, Samuk K. 2021. pixy: unbiased estimation of nucleotide diversity and divergence in the presence of missing data. Mol Ecol Resour 21:1359–1368. doi:10.1111/1755-0998.13326 33453139 PMC8044049

[88] Danecek P, Auton A, Abecasis G, Albers CA, Banks E, DePristo MA, Handsaker RE, Lunter G, Marth GT, Sherry ST, McVean G, Durbin R, 1000 Genomes Project Analysis Group. 2011. The variant call format and VCFtools. Bioinformatics 27:2156–2158. doi:10.1093/bioinformatics/btr330 21653522 PMC3137218

[89] Millard SP. 2013. EnvStats: an R package for environmental statistics. Springer New York.

[90] NCCLS. 2004. Method for antifungal disk diffusion susceptibility testing of yeasts; approved guideline. NCCLS document M44-A. NCCLS, Wayne, PA.

[91] Zupan J, Raspor P. 2008. Quantitative agar-invasion assay. J Microbiol Methods 73:100–104. doi:10.1016/j.mimet.2008.02.009 18358550

